# Expression of Concern for "Extracorporeal Shock Wave Therapy Versus Phonophoresis Therapy for Neck Myofascial Pain Syndrome: A Randomized Clinical Trial" [Anesth Pain Med. 2021;11(2):e112592]

**DOI:** 10.5812/aapm-144495

**Published:** 2024-01-20

**Authors:** Mahmood-Reza Alebouyeh

**Affiliations:** 1 Iran University of Medical Sciences, Tehran, Iran

The Editor-in-Chief of Anesthesia and Pain Medicine is issuing this Expression of Concern regarding the article titled "Extracorporeal Shock Wave Therapy Versus Phonophoresis Therapy for Neck Myofascial Pain Syndrome: A Randomized Clinical Trial" by Taheri et al., published in Anesthesia and Pain Medicine in 2021 (1).

This Expression of Concern has been prompted by the receipt of an anonymous communication raising concerns about the description of the shock wave therapy device used in the study. Specifically, the concern points to a discrepancy between the mentioned device in the article, the Dornier AR2, and the device name, the Duolith SD1, both of which are associated with focused extracorporeal shock wave therapy (fESWT). The anonymous correspondent has noted that these are two distinct devices, and the lack of clarity on which device was actually used in the study raises questions about the reproducibility of the research.

The accuracy and transparency of device descriptions in scientific research are essential for ensuring the replicability of studies by other researchers. Given the identified discrepancy and the importance of specifying the exact device used in fESWT research, we are taking this Expression of Concern seriously.

Until this matter is resolved, this Expression of Concern will remain attached to the article, ensuring that readers are aware of the concern raised and that the issue is being investigated with diligence and transparency.
